# Supplementary material for: The Systems Biology Research Tool: evolvable open-source software
Source: BMC Syst Biol. 2008 Jun 29;2:55. doi: 10.1186/1752-0509-2-55 (PMC2446383; doi:10.1186/1752-0509-2-55)
Supplement: Additional file 1 — SBRT Archive. An archive of the current version of the Systems Biology Research Tool. [file 1752-0509-2-55-S1.zip › sbrt-1.4.0/doc/developers_guide/api/sbrt/kernel/math/stat/proc/CorrelationAnalysis.html]

CorrelationAnalysis


|  |  |  |
| --- | --- | --- |
| **Overview** \| **Package** \| **Class** \| **Use** \| **Tree** \| **Deprecated** \| **Index** \| **Help** | |  |
| PREV CLASS   **NEXT CLASS** | **FRAMES**    **NO FRAMES**     **All Classes** |  |
| SUMMARY: NESTED \| FIELD \| CONSTR \| METHOD | DETAIL: FIELD \| CONSTR \| METHOD |  |


---


## sbrt.kernel.math.stat.proc Class CorrelationAnalysis<V,T extends CorrResult>

```
java.lang.Object
  sbrt.kernel.math.stat.proc.CorrelationAnalysis<V,T>
```

**Type Parameters:**: `V` - the variable type.: `T` - the correlation result type.

**All Implemented Interfaces:**: KernelProcess<T>

---

``` public class CorrelationAnalysis<V,T extends CorrResult> extends java.lang.Object implements KernelProcess<T> ```

This class is used to perform correlation experiments.

**Author:**
:   This class was written and documented by Jeremiah Wright
    while in the Wagner lab.

---

**Constructor Summary**

`CorrelationAnalysis(Correlator<V,T> correlator)`
  
          Constructs a new correlation experiment.


| **Method Summary** | |
| --- | --- |
| `void` | `run(ResultsManager<T> resultsManager)`             Computes the correlation statistic between the x and y values, and adds the results to the provided results manager. |
| `void` | `setXValues(java.util.Map<? extends V,java.lang.Double> xValues)`             Sets the x values. |
| `void` | `setYValues(java.util.Map<? extends V,java.lang.Double> yValues)`             Sets the y values. |

**Methods inherited from class java.lang.Object**

`clone, equals, finalize, getClass, hashCode, notify, notifyAll, toString, wait, wait, wait`

**Constructor Detail**

### CorrelationAnalysis

```
public CorrelationAnalysis(Correlator<V,T> correlator)
```

:   Constructs a new correlation experiment.

    **Parameters:**: `correlator` - the correlator with which all computations will be performed.


**Method Detail**

### setXValues

```
public void setXValues(java.util.Map<? extends V,java.lang.Double> xValues)
```

:   Sets the x values.

    :   **Parameters:**: `xValues` - variables mapped to their respective values.

---


### setYValues

```
public void setYValues(java.util.Map<? extends V,java.lang.Double> yValues)
```

:   Sets the y values.

    :   **Parameters:**: `yValues` - variables mapped to their respective values.

---


### run

```
public void run(ResultsManager<T> resultsManager)
```

:   Computes the correlation statistic between the x and y values,
    and adds the results to the provided results manager.

    :   **Specified by:**: `run` in interface `KernelProcess<T extends CorrResult>`
    :   **Parameters:**: `resultsManager` - the results manager to which the computed results are to be added.


---


|  |  |  |
| --- | --- | --- |
| **Overview** \| **Package** \| **Class** \| **Use** \| **Tree** \| **Deprecated** \| **Index** \| **Help** | |  |
| PREV CLASS   **NEXT CLASS** | **FRAMES**    **NO FRAMES**     **All Classes** |  |
| SUMMARY: NESTED \| FIELD \| CONSTR \| METHOD | DETAIL: FIELD \| CONSTR \| METHOD |  |


---
